# Combined Multi-Omics and Co-Expression Network Analyses Uncover the Pigment Accumulation Mechanism of Orange-Red Petals in *Brassica napus* L.

**DOI:** 10.3390/biology14060693

**Published:** 2025-06-13

**Authors:** Ledong Jia, Shengting Li, Chao Zhang, Lijun Zeng, Shulin Shen, Nengwen Yin, Huiyan Zhao, Zhanglin Tang, Cunmin Qu, Jiana Li, Zhiyou Chen

**Affiliations:** 1 Integrative Science Center of Germplasm Creation in Western China (CHONGQING) Science City and Southwest University, College of Agronomy and Biotechnology, Southwest University, Chongqing 400715, China; drjolly2021@swu.edu.cn (L.J.); lishengting0123@163.com (S.L.); zhh5201314@email.swu.edu.cn (L.Z.); ssl7159@email.swu.edu.cn (S.S.); nwyin80@126.com (N.Y.); zhaohuiyan@swu.edu.cn (H.Z.); tangzhlin@swu.edu.cn (Z.T.); drqucunmin@swu.edu.cn (C.Q.); 2 Guizhou Rapeseed Research Institute, Guizhou Academy of Agricultural Sciences, Guiyang 550006, China; 18083606406@163.com; 3 Chongqing Engineering Research Center for Rapeseed, Academy of Agricultural Sciences, Southwest University, Chongqing 400715, China

**Keywords:** *Brassica napus* L., flower color, transcriptome, metabolome, anthocyanin, WGCNA

## Abstract

Rapeseed (*Brassica napus* L.) has been cultivated as an ornamental plant in recent years. However, the metabolic and regulatory processes involved in pigment accumulation in. *B. napus* flowers are poorly understood. To address this knowledge gap, we conducted a multi-omics analysis of the orange-red-flowered ‘OrP’ and the yellow-flowered ‘ZS11’ rapeseed cultivars. The total anthocyanin content of ‘OrP’ petals was 5.420-fold and 3.345-fold higher than ‘ZS11’ petals at the S2 and S4 developmental stages, respectively. The red coloration of ‘OrP’ flowers resulted primarily from the presence of anthocyanin pigment derivatives. The up-regulated differentially expressed genes (DEGs) of four stages in ‘OrP’ were found to be significantly enriched in phenylpropanoid, flavonoid, and anthocyanin metabolism-associated GO and KEGG terms. Weighted Gene Co-expression Network Analysis (WGCNA) revealed that 51 DEGs were linked to anthocyanin metabolism, including several structural genes such as *BnaCHS*, *BnaF3H*, *BnaF3′H*, *BnaCHS*, *BnaDFR*, *BnaANS*, *BnaUGTs*, and the transcription factor (TF) genes *BnaHY5*, *BnaBBX22*, *BnaPIL1*, *BnaPAP2*, *BnaTT8*, *BnaTTG2*, and *BnaMYBL2*. Furthermore, we found that three main factors affecting the relative content of anthocyanins in petals were likely responsible for the fading of ‘OrP’ petals, namely the significantly down-regulated expression of genes (*BnaDFR*, *BnaANS*, *BnaPAP2*, *BnaTT8,* and *BnaTTG2*) related to anthocyanin biosynthesis, the significantly up-regulated expression of genes (*Bna.BGLUs*, *Bna.PRXs,* and *BnaMYBL2*) related to anthocyanin degradation or the negative regulation of anthocyanin biosynthesis, and the rapidly increasing petals area.

## Simple Summary

*Brassica napus* L. (rapeseed) is an important oil crop in the world and also, in recent years, an ornamental plant. To uncover the molecular mechanisms involved in pigment accumulation of the orange-red-flowered ‘OrP’, we evaluated the phenotypic, physiological, and metabolic differences between ‘OrP’ and the yellow-flowered ‘ZS11’. Transcriptomic and co-expression gene network analyses were performed to discover the key genes associated with anthocyanin metabolism in ‘OrP’ petals at four developmental stages. Furthermore, we found that three main factors affecting the relative content of anthocyanins in petals were likely responsible for the fading of ‘OrP’ petals.

## 1. Introduction

Rapeseed is an incredibly versatile crop which is grown to produce edible oil, forage, condiments, vegetables, and renewable energy. In addition, rapeseed fields have also gained popularity as tourist attractions. Over 20 Chinese agritourism destinations are known to feature scenic rapeseed fields, with 10 of these being well known for their large-scale rapeseed cultivation [1]. Flower color greatly influences the ornamental significance of plants, and has long fascinated both breeders and consumers [2,3]. However, rapeseed flowers are almost universally yellow [4], which can lead to visual fatigue during extended viewing. The scarcity of natural germplasm resources exhibiting flower colors other than yellow has, therefore, become a major obstacle for breeders looking to create new ornamental rapeseed varieties. To enhance the genetic diversity of cultivated rapeseed and to introduce novel flower colors, breeders have hybridized yellow-flowered rapeseed with other Brassicaceous species such as *Brassica alboglabra*, *Orychophragmus violaceus*, and *Raphanus* spp. [1,5,6,7]. Research has identified a few mutations impacting petal color in rapeseed, resulting primarily in golden yellow, orange-yellow, creamy white, and white flowers [7]. Through multiple rounds of selection involving self-pollination and crossbreeding, breeders have successfully obtained more than 10 different flower colors in rapeseed, including orange-red, pink, purple, red, deep red, and mottled [1,8].

In Brassicaceous plants, flower color is primarily determined by the assortment and concentration of pigments such as anthocyanins, carotenoids, and chlorophylls in the petals [7,9,10]. In the yellow-flowered *Brassica* species, a CACTA-like transposable element (TE) insertion was identified in the coding sequence of the carotenoid cleavage dioxygenase (CCD) 4 gene (*BnaC3.CCD4*). This insertion disrupts gene expression and prevents carotenoid degradation, resulting in a change from white to yellow flowers [5]. Additionally, zeaxanthin epoxidase gene (*Bna.ZEP*), which converts zeaxanthin to violaxanthin and antherxanthin, was found to change petals from yellow to orange. Knockout of this gene using the CRISPR/Cas9 technique leads to increased lutein accumulation in petal tissues [11]. Furthermore, the production of anthocyanin pigment 2 genes (*Ov.PAP2* and *BnaA07.PAP2*), which are homologous to *AtPAP2* in *Arabidopsis thaliana*, have been shown to significantly increase anthocyanin content in *B. napus* petals, thus changing their color from yellow to orange-red [6,10]. Anthocyanin and carotenoid metabolism is well characterized at both the molecular and genetic levels [12,13]. Petal color stability is also influenced by light, temperature, and pH, as well as the presence of oxidizing or reducing agents [14,15]. However, the regulatory dynamics of anthocyanin accumulation and the molecular mechanisms underlying the development of orange-red petals are not comprehensively described in *B. napus*.

In this work, in order to investigate the accumulation of anthocyanins driving changes in petal color, we conducted phenotypic, physiological, and metabolic studies on petals at different stages of petal development between two rapeseed varieties: orange-red-flowered ‘OrP’ and yellow-flowered ‘ZS11’. Additionally, we performed transcriptomic and co-expression gene network analyses at four developmental stages to discover the key genes associated with anthocyanin metabolism in petals. Our comprehensive analysis of the drivers of petal pigmentation in *B. napus* has helped to uncover the molecular mechanisms involved in anthocyanin metabolism. Furthermore, the key genes reported in this work will become useful genetic resources for the molecular breeding of ornamental rapeseed cultivars.

## 2. Materials and Methods

### 2.1. Cultivation and Sampling of Plant Materials

The yellow-flowered rapeseed cultivar ‘ZS11’ and orange-red-flowered rapeseed cultivar ‘OrP’ were grown at the Rapeseed Engineering Research Center experimental station of Southwest University in Beibei, Chongqing City, China (29°46′ N, 106°21′ E). The inbred line ‘OrP’ was first developed by Xiaonan Li’s group and represents a distant hybridization between a purple-flowered *Raphanus* spp. landrace and a yellow-flowered *B. napus* variety. Plants were grown in blocks, with each block consisting of ten rows and each row containing eight plants.

For metabolomic and transcriptomic analyses, petal samples were collected at each developmental stage from 30 haphazardly selected plants within the same block. Specifically, according to the changes in the petal character observed with the naked eye and the change laws of Δa colorimetric value determined by YS3060 Grating Spectrophotometer (Shenzhen Threenh Technology Co., Ltd., Shenzhen, China), petal samples were collected at the following developmental stages: 5 mm buds (B5P), in which the petal color changes from pale green to pale red; 8 mm buds (B8P), in which buds were at maximum length before flowering; open buds prior to petal spread (S1P); flowers with half-spread petals (S2P); flowers with newly spread petals (S3P); and flowers with fully spread petals (S4P). Each petal sample was frozen immediately in liquid nitrogen for storage at −80 °C.

### 2.2. Phenotypic Observation

Petal morphology was observed using an Olympus SZ61 stereomicroscope (Olympus, Tokyo, Japan). Chromaticity was evaluated with a YS3060 Grating Spectrophotometer using an 8 mm aperture size. Each assay was repeated three times. A white background was used as the white color reference. For the colorimetry analysis, the ΔE colorimetric value expresses the total difference in color, the Δb colorimetric value expresses yellow/blue color (+yellow, −blue), the Δa colorimetric value expresses red/green color (+red, −green), and the ΔL colorimetric value expresses brightness (+luminous, −dark).

### 2.3. Analysis of Pigment Content

To extract total anthocyanins from each sample, shredded petals (0.1 g) were combined with 2 mL of 80% acidified methanol (1% HCI, *v*/*v*). The solution was then incubated in darkness for 48 h at 4 °C with gentle shaking every 6 h until the petals lost their red coloration. Following extraction, the absorbance (A) of the supernatant (200 μL) was measured at 530 and 657 nm. The anthocyanin concentration (mg/g FW) was quantified using the formula (A530 − 0.25 × A657)/M [16,17], where M represents the fresh weight of the sample.

To extract the photosynthetic pigments (carotenoids and chlorophyll [a, b, total]) from each sample, shredded petals (0.1 g) were combined with 2 mL of 80% acetone. The solution was then incubated for 48 h in darkness at 37 °C with gentle shaking every 6 h until the petals were completely discolored. Following extraction, the absorbance (A) of the supernatant (200 μL) was measured at 470, 646, 652, and 663 nm. The contents of the different photosynthetic pigments were determined as follows:

CT = A652 × 1000/34.5(1)

Cx.c = (1000 × A470 − 3.27 × Ca − 104 × Cb)/229(2)

Cb = 20.13 × A646 − 5.03 × A663(3)

Ca = 12.21 × A663 − 2.81 × A646(4)

where CT is the combined content of chlorophyll b and a, Cx.c is the carotenoid content, Cb is the chlorophyll b content, and Ca is the chlorophyll a content [18,19].

The photosynthetic pigment concentration (mg/g FW) was determined according to the equation C × V/(M × 1000), where C is the photosynthetic pigment content, V is the volume, and M is the fresh weight [20]. Three biological replicates were used for each assay.

### 2.4. Metabolomic Analysis

To compare the anthocyanin and carotenoid metabolites in ‘OrP’ and ‘ZS11’ petals at the S2 and S4 developmental stages, we carried out ultra-high performance liquid chromatography–electrospray ionization–tandem mass spectrometry (UPLC-ESI-MS/MS) (SHIMADZU CBM30A; Shimadzu Corporation, Kyoto, Japan). Applied Biosystems 4500 QTRAP; AB SCIEX company, Foster City, CA, USA). The system consisted of a Shimadzu Shim-pack CBM30A UFLC system coupled with an Applied Biosystems 4500 Q TRAP MS. Analyses were carried out using a combination of the MetWare database (Wuhan Metware Biotechnology, Wuhan, China) and other publicly available data sources [7]. In addition, the anthocyanin metabolites of ‘OrP’ and ‘ZS11’ petals at the B5, B8, S1, and S3 developmental stages were analyzed using an UPLC-HESI-MS/MS system (Ultimate 3000/Q Exactive; Thermo Fisher Scientific Inc., Bremen, Germany). The chromatographic-grade methanol was used as the blank control sample. For the detailed procedure, please refer to our previously published article [4]. An in-house database was used for data analysis [4]. Each experiment was conducted with three independent biological replicates.

### 2.5. Transcriptomic Analysis

Total RNA was extracted from ‘OrP’ and ‘ZS11’ petals at the B5, B8, S1, and S3 developmental stages with an EZ-10 DNAaway RNA Mini-Preps Kit (Shanghai Sangon Bioengineering, Shanghai, China). Preparation of the RNA-seq library and sequencing were carried out by Novogene (Beijing, China). Sequencing was carried out on an Illumina HiSeq 4000 platform, generating 150 bp paired-end reads. The raw transcriptomic data have been deposited (accession number PRJCA001841) in the Genome Sequence Archive (GSA) of the China National Center for Bioinformation/Beijing Institute of Genomics (BIG) Data Center, Chinese Academy of Sciences (https://bigd.big.ac.cn/gsa, accessed on 17 November 2023) [21,22]. Adapters and low-quality sequences were removed prior to downstream analyses. The *B. napus* ‘ZS11’ reference genome was obtained from the BnPIR database (http://cbi.hzau.edu.cn/bnapus/, accessed on 14 January 2020) [23,24]. Clean data were mapped to the reference genome using HiSat 2.0.4 [25]. Gene expression was quantified using Cufflinks 2.2.1 [26]. For each gene, the Fragments Per Kilobase Million (FPKM) value was determined based on the gene length and the count of mapped reads. Differentially expressed genes (DEGs) were screened using the R package DESeq2 1.12.4 [27], with three biological replicates per sample. DEGs were considered statistically significant if they met the following criteria: adjusted *p*-value (FDR) < 0.001 and absolute fold-change (FC) > 4 [7]. Gene Ontology (GO) and Kyoto Encyclopedia of Genes and Genomes (KEGG) enrichment analyses were conducted using the TBtools v1.113 toolkit [28], with an adjusted *p*-value < 0.05 indicating significance.

### 2.6. Co-Expression Network Analysis of Metabolome and Transcriptome

To perform Weighted Gene Co-expression Network Analysis (WGCNA), we utilized the WGCNAshiny tool of TBtools [28]. Specifically, we combined chromaticity data, differential anthocyanin data, and differential gene expression data. After setting an expression threshold and removing 1 outlier, 7736 genes with an average FPKM > 5 were retained. The soft thresholding method allowed us to determine an appropriate power value (15), ensuring a scale-free topology fit index (scale-free R^2^ = 0.74 > 0.7) and an optimal mean connectivity (Figure S3). The min-module-size was 30 and the module-cuttree-height was 0.25, and the remaining parameters utilized default settings. The networks were visualized using Cytoscape 3.91 [29].

### 2.7. Quantitative Real-Time PCR Validation

We validated the expression patterns of 26 DEGs associated with the anthocyanin metabolic pathway using real-time quantitative PCR (RT-qPCR). All gene-specific primers can be found in Table S1. cDNA synthesis and PCR cycling were carried out following previously described methods [7]. Relative gene expression was quantified according to the 2^−ΔΔCt^ method, with *BnACTIN7* utilized as the internal control [30]. All assays were conducted with three biological replicates, and the average values were used for downstream analyses.

### 2.8. Statistical Analysis

The statistical analysis was performed using SPSS 15.0 software (one-way ANOVA with Tukey’s assay; SPSS Inc., Chicago, IL, USA), and all analyses were conducted in triplicate. The data were expressed as the mean ± standard deviation (SD) of three replicates, and differences were considered significant at the *p* < 0.05 level.

## 3. Results

### 3.1. Anthocyanins and Carotenoids Contribute to the Color of ‘OrP’ Petals

**Figure 1:**
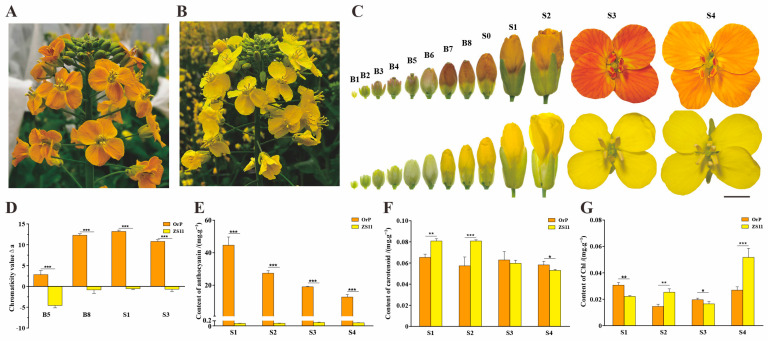
The morphological characteristics and pigment contents of the ‘OrP’ and ‘ZS11’ flowers at different developmental stages. (**A**,**B**) Morphological map of the orange-red-flowered rapeseed ‘OrP’ and the yellow-flowered rapeseed ‘ZS11’. (**C**) Morphological appearance of the petals of ‘OrP’ and ‘ZS11’ at different developmental periods. The stages are as follows: B1 to B8, the maximum length of the buds ranging from 1 to 8 mm; S0, petals just exposed from the sepals; S1, petals exposed from open buds before spreading; S2, petals half spread; S3, petals just fully spread; S4, petals fully spread to the maximum size. Scale bar: 5 mm. (**D**) The chromaticity values Δa (red-green color) of the petals in different stages for ‘OrP’ and ‘ZS11’. (**E**–**G**) The total anthocyanin, carotenoid, and chlorophyll contents of the petals in different stages for ‘OrP’ and ‘ZS11’. Error bars indicate the standard deviation of three independent replicates. Asterisks (*, **, or ***) denote significant differences at *p* < 0.05, *p* < 0.01, and *p* < 0.001, respectively.

The most noticeable morphological difference between ‘OrP’ and ‘ZS11’ was petal color (Figure 1A,B). Both ‘OrP’ and ‘ZS11’ exhibited green petals between the B1 and B4 developmental stages. However, between B5 and S4, ‘OrP’ exhibited petals with varying degrees of red on a yellow background, ranging from light pink to orange-red. In contrast, the petals of ‘ZS11’ were various shades of yellow, ranging from ivory to yellow (Figure 1C).

The ΔE colorimetric values of ‘OrP’ petals were significantly higher (*p*-value < 0.05) than those of ‘ZS11’ from stage B5 to stage S1 (Figure S1A). Although both varieties exhibited negative values, the ΔL colorimetric values of ‘OrP’ petals were significantly lower than those of ‘ZS11’, indicating that they were visually darker (Figure S1B). The Δa colorimetric values of ‘OrP’ petals were all positive, indicating a reddish color compared with the white background. Meanwhile, the Δa values of ‘ZS11’ petals were all negative, indicating a greenish color (Figure 1D). The Δb colorimetric values of ‘ZS11’ petals were significantly higher than those of ‘OrP’, indicating a yellowish color compared with ‘OrP’ (Figure S1C).

Spectrophotometry was utilized to measure the relative contents of different pigments in the petals. The total carotenoid concentration of ‘OrP’ was found to be significantly lower than that of ‘ZS11’ between the stages of S1 and S2, but higher between S3 and S4 (Figure 1E). The total anthocyanin concentration of ‘OrP’ petals was significantly higher than that of ‘ZS11’ from stage S1 to S4 (Figure 1F). The concentrations of total chlorophyll and chlorophyll a in ‘OrP’ petals were significantly greater than those of ‘ZS11’ between stages S1 and S3. However, the concentrations of chlorophyll a/b and total chlorophyll were significantly lower between stages S2 and S4 (Figure 1G and Figure S1D,E).

The carotenoid extraction solutions of ‘OrP’ and ‘ZS11’ petals were yellow, and the anthocyanin extraction solution of ‘OrP’ petals was purple, while that of the ‘ZS11’ petals was colorless (Figure S1G,H). Taken together, these data suggest that the primary pigments underlying the color of ‘OrP’ petals are anthocyanins and carotenoids.

### 3.2. Pigment-Associated Metabolomic Profiling of ‘OrP’ and ‘ZS11’ Petals

The anthocyanins- and carotenoids-targeted metabolomics were performed using the petal samples at the S2 and S4 stages in ‘OrP’ and ‘ZS11’; the results indicated that the total anthocyanin concentration of ‘OrP’ petals was 5.420 times higher than that of ‘ZS11’ at the S2 stage and 3.345 times higher at the S4 stage (Table S2). In addition, ‘OrP’ petals contained significantly higher contents of the three main anthocyanins contributing to reddish coloration (paeoniflorin O-hexoside, cyanidin 3-O-glucoside, and cyanidin O-syringic acid) than ‘ZS11’ petals. Specifically, the contents of these anthocyanins were 174.257, 24.315, and 2293.578 times higher in ‘OrP’ petals than in ‘ZS11’ petals at the S2 stage, and 188.841, 29.746, and 1968.504 times higher at the S4 stage, respectively (Table S2). On the other hand, the contents of total carotenoids in ‘OrP’ petals were 1452.441 μg/g and 1423.386 μg/g at the S2 and S4 stages, respectively (Table S3). In comparison to the content of total carotenoids previously reported for ‘ZS11’ petals [7], ‘OrP’ petals exhibited 0.880 and 0.555 times lower contents of total carotenoids than ‘ZS11’ at stages S2 and S4, respectively (Table S3). Similar to the previous study, lutein and zeaxanthin were identified as the main yellow pigments in ‘ZS11’ petals [7]. The total contents of lutein and zeaxanthin in ‘OrP’ petals were 1.927 and 0.948 times higher than ‘ZS11’ at the S2 and S4 stages, respectively.

**Figure 2:**
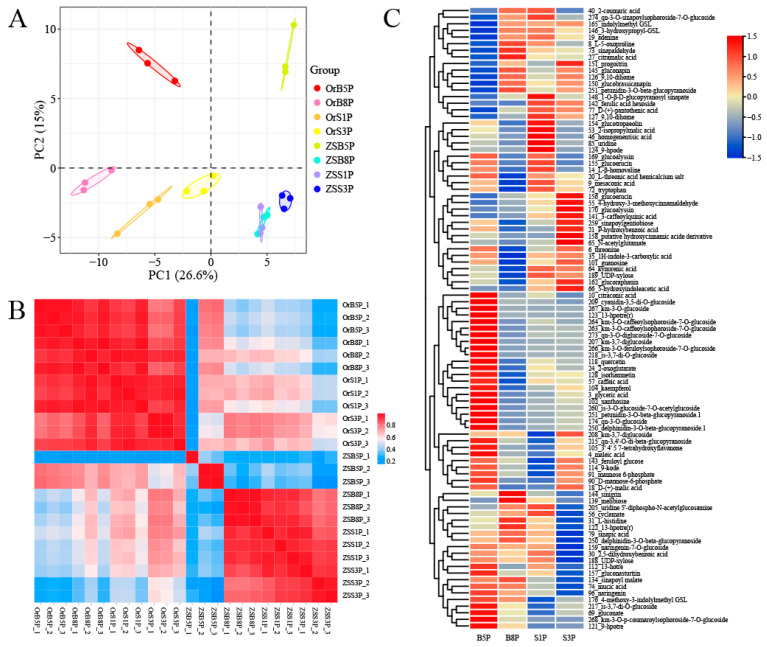
The metabolome quality and differential metabolite analysis. (**A**) Principal component analysis, PC1 and PC2 are plotted on the x- and y-axes, respectively. (**B**) Correlation heat map showing that ‘OrP’ and ‘ZS11’ separated significantly. (**C**) Heatmap of the differentially accumulated metabolites, up- and down-regulated metabolites (red and blue color) of the values of log_2_ (fold change).

The untargeted metabolomics analysis was conducted using the ‘OrP’ and ‘ZS11’ petals at the B5, B8, S1, and S3 stages through UPLC-HESI-MS/MS. The principal component analysis (PCA) showed that the first principal component could explain 26.6% of the total variance and separate the ‘OrP’ and ‘ZS11’ petals by color. The second principal component explained 15% of the total variance and distinguished petal samples based on the B5 and B8-S3 stages (Figure 2A). The results of the correlation analysis between the samples indicate that petals at different stages had good biological reproducibility (Figure 2B). We identified 154 metabolites in the petals of ‘OrP’ and ‘ZS11’ at 4 stages and a total of 94 significantly different accumulated metabolites (DAMs) (Table S4), 19 of which were commonly identified across 4 petal stages (Figure S2). Based on fold changes in the differential metabolites, the DAMs heatmap was produced to visualize the degree of metabolites, which could clearly show the changes at 4 stages between ‘OrP’ and ‘ZS11’ petals (Figure 2C). These DAMs included five of six identified anthocyanin metabolites: cyanidin-3,5-di-O-glucoside, petunidin-3-O-beta-glucopyranoside, petunidin-3-O-beta-glucopyranoside.1, delphinidin-3-O-beta-glucopyranoside, and delphinidin-3-O-beta-glucopyranoside.1, and they were accumulated significantly in ‘OrP’ petals (Table S4).

These data suggested that the primary pigments underlying the red coloration of ‘OrP’ petals maybe the cyanidin and paeoniflorin derivatives, while lutein and zeaxanthin underlie the yellow coloration of ‘OrP’ and ‘ZS11’ petals.

### 3.3. Pigment-Associated Transcriptomic Profiling of ‘OrP’ and ‘ZS11’ Petals

RNA-seq analysis was performed on ‘OrP’ petals at four major stages of petal development (B5, B8, S1, and S3), and the main characteristics of the cDNA libraries and RNASeq data are outlined in Table S5. After the removal of low-quality reads, between 21,437,135 and 31,832,494 clean reads were obtained, with a G + C percentage ranging from 46% to 47%, and a Q30 exceeding 97.33%. These clean sequences were then mapped to the *B. napus* ‘ZS11’ reference genome [23], resulting in a total mapping ratio ranging from 91.31% to 92.83%. The clean reads with the best score match ratio (>86.59%) were further analyzed for FPKM values to compare the gene expression differences among samples.

**Figure 3:**
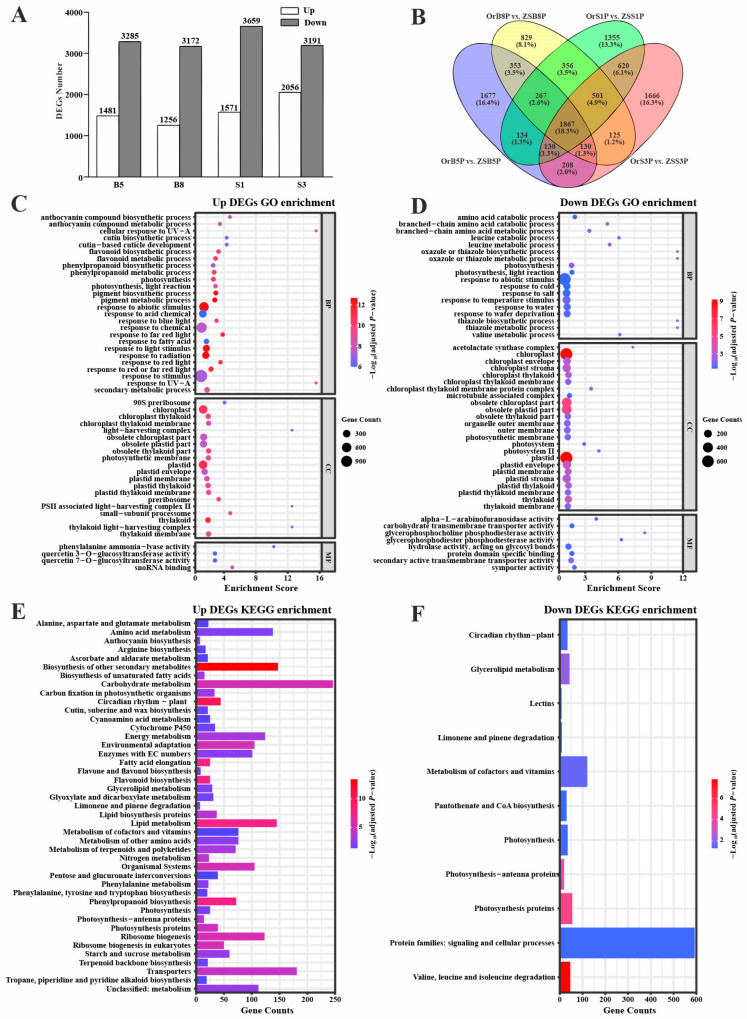
The number of DEGs and GO and KEGG enrichment analyses in different compared combination groups of ‘OrP’ and ‘ZS11’. (**A**) The number of up- and down-regulated DEGs in different comparison groups. (**B**) Venn diagram of DEGs in different comparison groups. (**C**,**D**) GO enrichment analysis of the up- and down-regulated DEGs. The size of each point shows the number of genes in the term. BP, biological process; CC, cellular component; MF, molecular function. (**E**,**F**) KEGG enrichment analysis of the up- and down-regulated DEGs.

In comparison to previously reported transcriptomic data of ‘ZS11’ petals at the B5, B8, S1, and S3 stages [7], we identified a total of 10,218 (3996 up-regulated and 6292 down-regulated) DEGs, including 4766 (1481 up-regulated and 3285 down-regulated) at the B5 stage, 4428 (1256 up-regulated and 3172 down-regulated) at the B8 stage, 5230 (1571 up-regulated and 3659 down-regulated) at the S1 stage, and 5247 (2056 up-regulated and 3191 down-regulated) at the S3 stage (Figure 3A, Table S6). Additionally, 1867 DEGs were found to be common to all four comparison groups (OrB5P vs. ZSB5P, OrB8P vs. ZSB8P, OrS1P vs. ZSS1P, and OrS3P vs. ZSS3P) (Figure 3B).

GO and KEGG enrichment analyses were performed on the identified DEGs. The 3996 up-regulated DEGs were enriched in photosynthesis, the phenylpropanoid biosynthetic process, the anthocyanin biosynthetic process, and the flavonoid biosynthetic process. In addition, GO biological processes related to light signaling were significantly enriched (Figure 3C,D). The 6292 down-regulated DEGs were enriched in photosynthesis for both GO biological processes and KEGG pathways (Figure 3E,F). Furthermore, the GO biological processes of response to abiotic stimulus (GO:0009628), including temperature (GO:0009266), drought (GO:0009414), salt (GO:190207), and cold (GO:0009409), were also enriched (Figure 3E). Although there were more down-regulated than up-regulated DEGs (Figure 3A), the enrichment results of the up-regulated DEGs align closely with the phenotype of ‘OrP’ petals, which exhibited a higher anthocyanin content.

### 3.4. WGCNA Reveals Key Genes Associated with Anthocyanin Metabolism in Rapeseed

**Figure 4:**
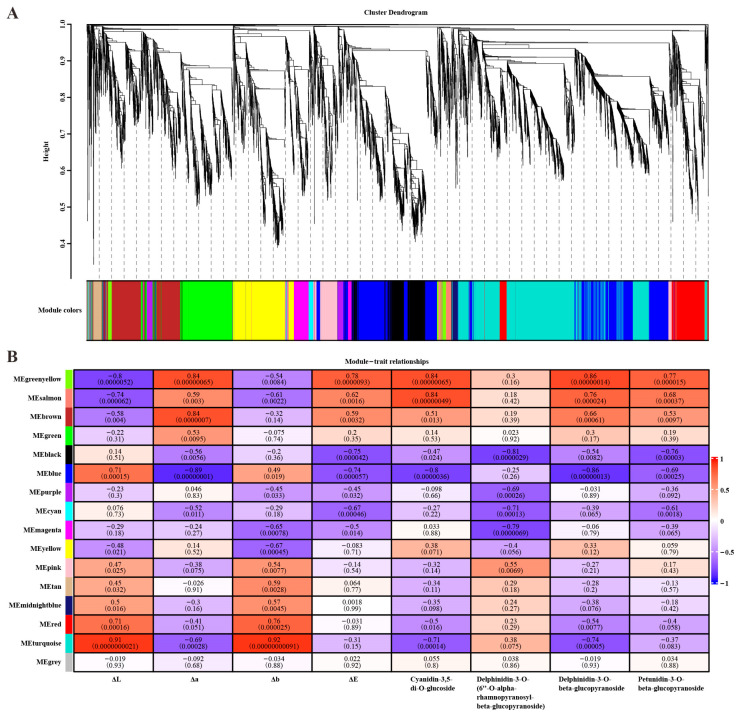
WGCNA of the anthocyanin contents and colorimetric values of the petals in ‘OrP’ and ‘ZS11’. (**A**) The hierarchical cluster tree showing the identified co-expression modules. (**B**) The correlations of the samples with WGCNA modules. Red and blue scale indicate positive and negative correlations.

WGCNA was performed, combining the expression of 7736 genes with an average FPKM value > 5 (excluding the outlier sample of OrS3P.3), the colorimetric values of petals, and the relative contents of cyanidin, delphinidin, and petunidin glucoside derivatives in ‘OrP’ and ‘ZS11’ petals (Table S7). A total of 15 WGCNA modules were obtained (Figure 4A), with the exception of the gray module which did not cluster with any other modules. Each module contained between 49 and 1263 genes, including between 2 and 91 transcription factors (TFs), and the module membership (MM) values for these modules were all >0.8 (Figure S4A). Among these, nine modules exhibited positive or negative correlations with anthocyanin content and/or colorimetric values, and these included the greenyellow, salmon, brown, black, blue, cyan, yellow, red, and turquoise modules, all of which exhibited module–trait relationship values > 0.6 (Figure 4B).

For each module, we conducted GO and KEGG enrichment analyses using the genes from the module eigengene. Interestingly, we found that the phenylpropanoid, flavonoid, and anthocyanin biosynthetic processes were significantly enriched in both the GO and KEGG results of the yellow module (Tables S8 and S9). A weight threshold of 0.45 was set to ensure a sufficient correlation between genes in the network. As a result, we identified 51 DEGs associated with the phenylpropanoid, flavonoid, and anthocyanin biosynthetic processes. Notably, these genes were correlated with a high anthocyanin content in ‘OrP’ petals (Table S8). In addition, the co-expressed genes in the network were significantly associated with lipid metabolism, wax metabolism, carbohydrate metabolism, growth and development, cell wall formation, photosynthesis, and various metabolic processes (Figure S4B).

### 3.5. Exploration of Anthocyanin Metabolism in B. napus

**Figure 5:**
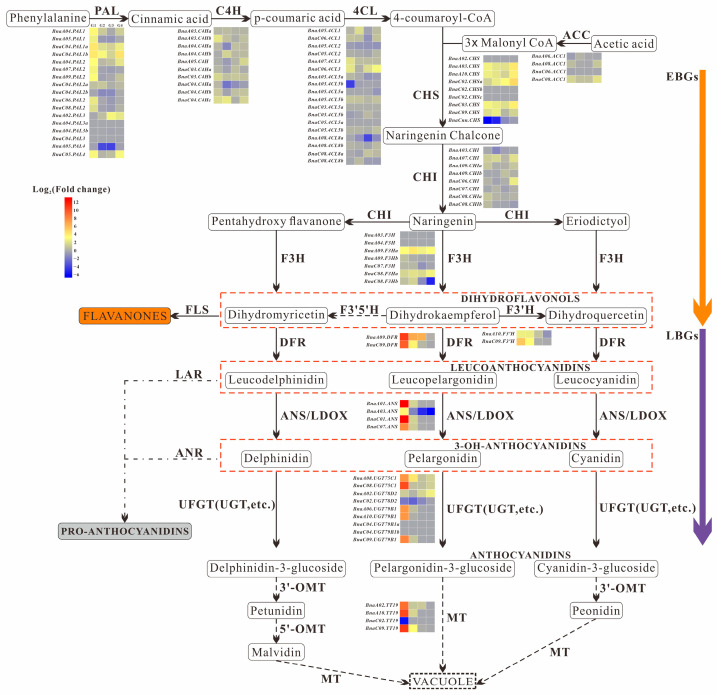
The metabolic pathway analysis of structural genes involved in anthocyanin biosynthesis. Red to blue scale indicate up- and down-regulation. G1–G4: Different compared combination groups of ‘OrP’ and ‘ZS11’ during B5, B8, S1, and S3 stages, respectively. EBGs, early biosynthetic genes; LBGs, late biosynthetic genes; PAL, Phenylalanin ammonialyase; C4H, Cinnamate 4-hydroxy-lase; 4CL, 4-coumarate CoA ligase; ACC, Acetic acid-CoA carboxylase; CHS, chaleone syn-thase; CHI, Chaleone isomerase; F3H, Flavanone 3-hydroxylase; F3′H, Flavonoid 3′-hydroxylase; F3′5′H, Flavanone 3′5′-hydroxy-lase; FLS, Flavonol synthase; DFR, Dihydroflavonol 4-reductase; ANS, Anthocyanin synthase; LAR, Leucoantho cyanidin reductase; ANR, Anthocyanidin reductase; UFGT, UDPG-flavonoid-3-O-glycosyltranferase; MT, Methylferase; 3′-OMT, 3′-O-Methyltransferase; 5′-OMT, 5′-O-Methyltransferase.

**Figure 6:**
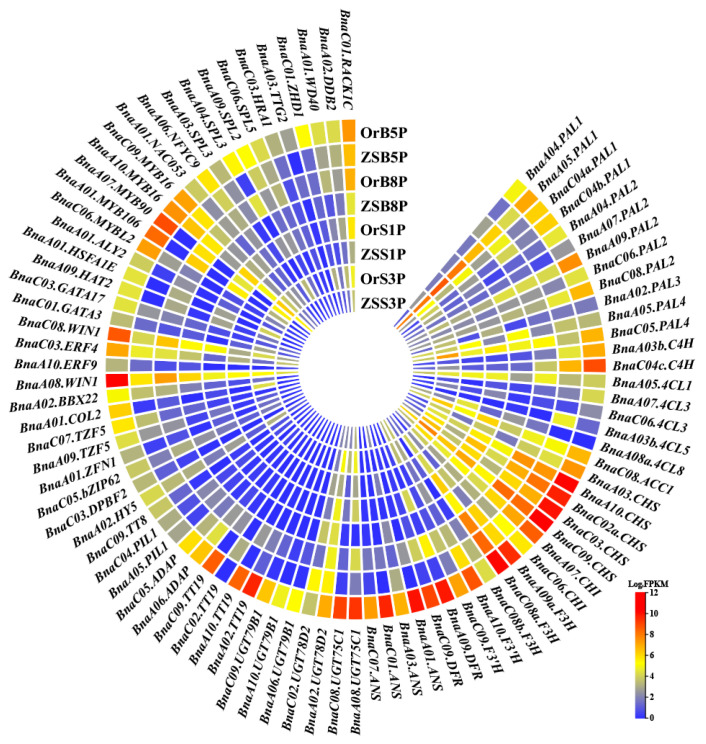
The expression profile of the structural genes and transcript factor genes involved in anthocyanin biosynthesis. Clustering heat map showing the abundance of genes by log_2_ of the FPKM values, where different colors from blue to red show gene expression level differences, with higher expression colored red and lower expression colored blue.

We identified 94 genes associated with anthocyanin metabolism from the rapeseed reference genome [23], and the expression patterns of all 94 genes are illustrated in Figure 5. Among these, 66 genes were found to be expressed with FPKM values > 5 (Figure 5), and 49 exhibited significant differential expression between ‘OrP’ and ‘ZS11’ during at least one developmental stage (Figure 5 and Figure 6).

Regarding phenylpropanoid metabolism, 13 out of 17 *Bna.PAL* genes were clearly expressed in both ‘OrP’ and ‘ZS11’ petals. Additionally, 12 genes showed significantly differential expression. Although 8 homologous *Bna.C4H* genes were clearly expressed, only 2 of them were found to be differentially expressed. In addition, 10 out of 18 *Bna.4CL* genes were clearly expressed, 5 of which exhibited differential expression.

For flavonoid metabolism, only 1 *Bna.ACC1* gene exhibited differential expression. Among the early biosynthetic genes (EBGs), 5 out of 9 *Bna.CHS* genes were clearly and differentially expressed, while 6 out of 8 *Bna.CHI* genes were clearly expressed, 2 of which showed differential expression. Finally, 3 out of 7 *Bna.F3H* genes and 2 *Bna.F3′H* genes exhibited clear and significant differential expression.

Regarding anthocyanin metabolism, all 17 late biosynthetic genes (LBGs) (with the exception of 2 *Bna.UGT79B1* genes) exhibited differential expression. Most of these genes were significantly up-regulated during the B5 stage, suggesting their involvement in anthocyanin biosynthesis or transport in ‘OrP’ petals. Overall, these findings serve as a valuable reference for further research on anthocyanin metabolism in *B. napus* petals.

### 3.6. Transcription Factors Contributing to Anthocyanin Metabolism in Rapeseed

Using the *B. napus* multi-omics information resource (BnIR) rapeseed TF database (https://yanglab.hzau.edu.cn/BnIR/TF, accessed on 24 April 2021) [31], we identified 39 TF genes in the yellow module, which may transcriptionally activate or repress anthocyanin metabolism in *B. napus*. Most of these genes exhibited similar expression profiles, with significant up-regulation in ‘OrP’ petals compared with ‘ZS11’ petals at different developmental stages (Figure 6).

Anthocyanin biosynthesis is regulated by the MBW transcriptional activation complex which consists of a WD-repeat (WDR) protein, bHLH, and R2R3-MYB TFs. The MBW complex directly interacts with the promoters of anthocyanin metabolism genes [32]. By studying homologous genes in *A. thaliana*, it was found that the *BnaA07.MYB90* gene (also known as *production of anthocyanin pigment 2* [*PAP2*]) encodes a R2R3-type MYB TF which positively regulates anthocyanin accumulation [33]. Compared with ‘ZS11’, *BnaA07.MYB90* was significantly up-regulated in ‘OrP’ petals from stage B5 to stage S3, suggesting that *BnaA07.MYB90* may play a central role in anthocyanin biosynthesis (Figure 6). On the other hand, the *BnaC06.MYBL2* gene was up-regulated in ‘OrP’ petals at the B5 stage, but its expression decreased after stage B8 compared with ‘ZS11’ (Figure 6). This gene is homologous to *At.MYBL2*, which represses anthocyanin accumulation by competing with the R2R3-MYBs PAP1 and PAP2 to form a complex with TT8 [34].

Three bHLH TF genes were identified in this study: two PIL1 genes (*BnaA05.PIL1* and *BnaC04.PIL1*) encoding a novel MYC-related bHLH PIF TF [35], and one *BnaC09.TT8* gene. The protein encoded by *BnaC09.TT8* acts in concert with TTG1, PAP1, and TT1 to regulate anthocyanin biosynthesis [36,37]. The expression of *BnaA05.PIL1* and *BnaC04.PIL1* was up-regulated in ‘OrP’ petals from stage B5 to stage B8, although these genes were not expressed at S1 or S3. The *BnaC09.TT8* gene was only up-regulated in ‘OrP’ petals at the B5 stage and was not expressed from stage B8 to stage S3 (Figure 6).

Three WD40 genes were found to be up-regulated in ‘OrP’ petals at all four developmental stages (Figure 6). Among these, *BnaA01.WD40* (*BnaA01G0379300ZS*) is homologous to *AT3G10530* and may be associated with the formation of E3 ubiquitin ligase [38]. *BnaA02.WD40* (*BnaA02G0108900ZS*) is homologous to *AT5G58760* and encodes the DDB1a-interacting protein, DDB2, which is essential for UV-B tolerance and genomic integrity [39]. Similarly, *BnaC01.WD40* (*BnaC01G0407400ZS*) is homologous to *AT3G18130* and encodes a protein which functions in shuttling activated protein kinase C to a variety of subcellular locations. Additionally, it may serve as a scaffold by physically interacting with an array of proteins [40].

Interestingly, the gene *BnaA02.HY5* (*BnaA02G0039400ZS*) was significantly up-regulated and is known to mediate anthocyanin accumulation under blue and far-red light, but not under red light or darkness [41,42]. Additionally, *BnaA02.BBX22* (*BnaA02G0232400ZS*) encodes a light-regulated zinc finger protein 1 (LZF1). These findings suggest that anthocyanin biosynthesis in ‘OrP’ petals may be regulated by the light environment [43,44]. Previous research reports that the WRKY TF gene *BnaA03.TTG2* (*BnaA03G0178600ZS*) co-regulates anthocyanin biosynthesis alongside the MBW complex and is associated with proanthocyanidin synthesis and seed color [45,46]. Interestingly, this gene was found to be up-regulated in ‘OrP’ petals at the B5 and B8 stages (Figure 6).

Four ERF TF genes were significantly up-regulated in ‘OrP’ petals. These genes, *BnaA08.WIN1* (*BnaA08G0268600ZS*) and *BnaC08.WIN1* (*BnaC08G0233100ZS*), are homologous to *AtWIN1* (*AT1G15360*). Additionally, *BnaA10.ERF9* (*BnaA10G0021700ZS*) is homologous to *AtERF9* (*AT5G44210*), and *BnaC03.ERF4* (*BnaC03G0795800ZS*) is homologous to *AtERF4* (*AT3G15210*). These ethylene-responsive TFs may mediate anthocyanin biosynthesis through the ethylene signaling pathway [47,48,49].

**Figure 7:**
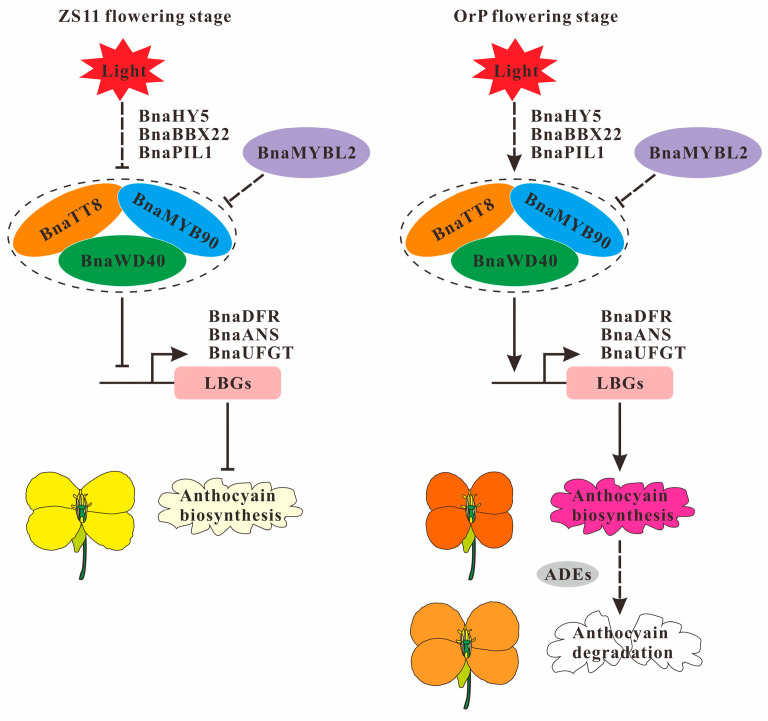
Working model of anthocyanin biosynthesis in ‘OrP’ and ‘ZS11’ petals. The arrows and blunt-ended lines indicate activation and repression, respectively. LBGs, late biosynthetic genes; ADEs, anthocyanin degradation enzymes.

Our findings indicate that anthocyanin biosynthesis in rapeseed is likely regulated by light through the involvement of *BnaA02.HY5*, *BnaA02.BBX22*, *BnaA05.PIL1*, and/or *BnaC04.PIL1*. Additionally, *BnaA07.MYB90* may control anthocyanin accumulation in ‘OrP’ petals. This gene could potentially interact with BnaC09.TT8 and/or Bna.WD40, which are both central aspects of the MBW complex. On the other hand, BnaC06.MYBL2 may inhibit anthocyanin accumulation by destabilizing the MBW complex (Figure 7).

### 3.7. Quantitative Real-Time PCR Validation of Anthocyanin Metabolism Genes

**Figure 8:**
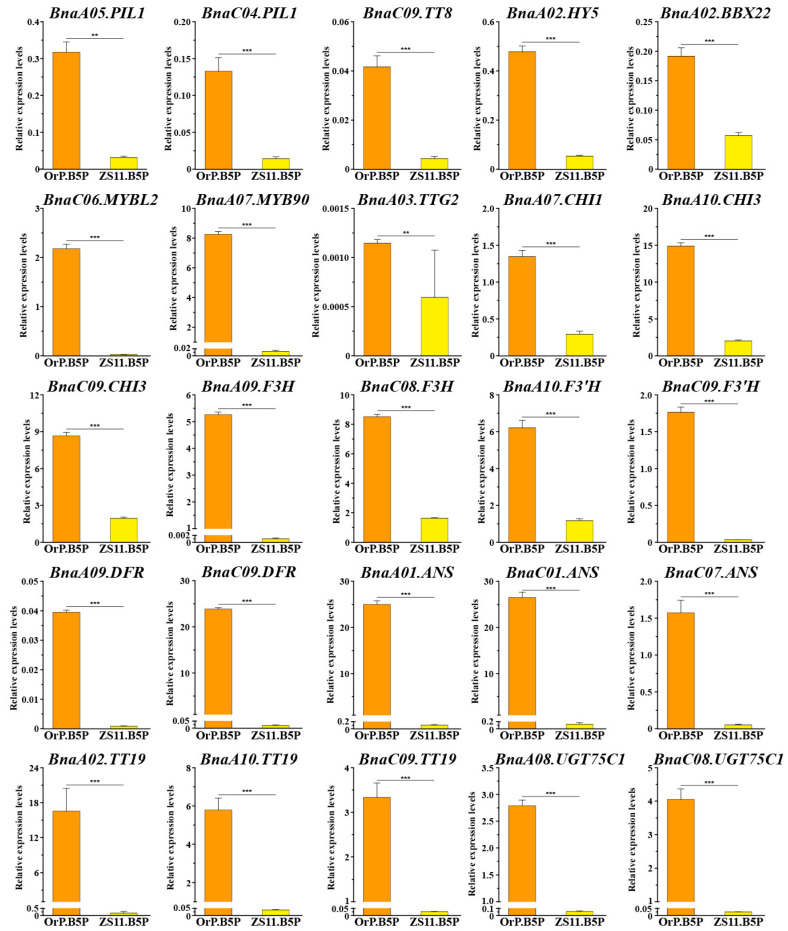
Quantitative real-time PCR (qRT-PCR) validation of the structural genes and candidate transcript factors involved in anthocyanin biosynthesis. Error bars indicated the standard deviation of three independent replicates. Asterisks (**, ***) denoted significant differences at *p* < 0.01 and *p* < 0.001, respectively.

We selected 26 key DEGs associated with anthocyanin metabolism for qRT-PCR validation of their expression patterns at the B5 stage. All primers used for this analysis are listed in Table S1. In all, 25 anthocyanin-associated genes were significantly up-regulated in ‘OrP’ petals compared with ‘ZS11’ petals (Figure 8). These genes included 8 TFs and 17 structural genes. The *BnaA03.ANS* gene was not included likely due to primer specificity. The patterns of expression obtained by qRT-PCR were largely consistent with the RNA-seq results, indicating that the transcriptomic data were reliable and reproducible.

## 4. Discussion

### 4.1. Formation of Orange-Red Petal Color in ‘OrP’ Rapeseed

Petal color heavily influences the ornamental value of plants, including rapeseed. Compared with other Brassicaceous species such as *O. violaceus* and radish, rapeseed petals exhibit a limited range of colors. Anthocyanin biosynthesis has been generally well characterized in plants [50,51]. However, although petal color is mainly influenced by anthocyanin accumulation in *B. napus* [4,10], the mechanisms responsible for the regulation of anthocyanin metabolism are poorly understood in rapeseed. To address this knowledge gap, we performed metabolomic and transcriptomic analyses on orange-red-flowered ‘OrP’ and yellow-flowered ‘ZS11’ rapeseed cultivars. Specifically, we analyzed the variation in petal coloration by comparing the contents of total anthocyanins, total carotenoids, and total chlorophylls between ‘OrP’ and ‘ZS11’. Significant differences were observed in the concentrations of these three pigment types in petals at different developmental stages (Figure 1E–G). The results suggest that the orange-red color of ‘OrP’ petals is mainly the result of anthocyanins and carotenoids, while the yellow color of ‘ZS11’ petals is the result of only carotenoids (Figure 7).

The metabolomic analysis of ‘OrP’ and ‘ZS11’ petals revealed that the main pigments affecting petal color were cyanidin and paeoniflorin derivatives, which exhibit a red hue. Anthocyanin biosynthesis involves flavonoid 3′5′-hydroxylase (F3′5′H), which leads to the production of delphinidin-based anthocyanins (including the delphinidin and petunidin derivatives) which typically exhibit violet and blue colors [6,52]. The delphinidin and petunidin derivatives were also detected in both ‘OrP’ and ‘ZS11’ (Tables S2 and S4). Previous studies have identified delphinidin-based anthocyanins in other *Brassica* species, such as purple bok choy [53] and purple heading Chinese cabbage [54], as well as in *B. napus* [6]. However, research suggests that the *A. thaliana* and *Brassica* genomes do not appear to contain homologous *F3′5′H* genes [6,55]. Therefore, *Brassica* genomes may harbor isoenzymes with similar catalytic activity to F3′5′H, although they have not yet been identified.

### 4.2. Mechanism of Petals Fading in ‘OrP’ Rapeseed

We observed that the red color of ‘OrP’ petals gradually faded following exposure between the S1 and S4 stages. Notably, the petals appeared significantly lighter at the S4 stage compared with the S1 stage (Figure 1C). Concurrently, as the petals grew and developed, their surface area gradually increased (Figure S1F). Conversely, the colorimetric values of Δa and the total anthocyanin concentration decreased gradually from stage S1 to stage S4 (Figure 1D,E). These findings suggest that the decrease in the relative anthocyanin content resulted in petals color fading.

The expression of most LBGs, including *Bna.DRF* and *Bna.ANS*, were significantly up-regulated in ‘OrP’ petals at the B5 stage (Figure 5, Figure 6 and Figure 7). However, their expression decreased at the B8 stage and was almost no longer detectable after stage S1 (Figure 6). These results indicate that anthocyanin biosynthesis may gradually diminish starting at the B8 stage. In addition, the expression pattern of *Bna.MYBL2* was similar to those of *Bna.DRF* and *Bna.ANS* (Figure 6). It is worth noting that *Bna.MYBL2* has been reported as the major negative regulator of anthocyanin metabolism [34].

To further clarify the molecular mechanism responsible for the fading of ‘OrP’ petals, we examined the expression of several genes encoding anthocyanin degradation enzymes (ADEs). These enzymes, including β-glucosidase (BGLU) [56], peroxidase (POD, PER, or PRX) [57,58,59], and polyphenol oxidase (PPO) [60], have been implicated in anthocyanin degradation in litchi, blood orange, rose, and *Brunfelsia calycina* [51]. Interestingly, no PPOs have been discovered in *A. thaliana* [61], although the homologous enzyme laccase (LAC) is responsible for the catalytic reaction [62]. The rapeseed ADE genes, which are homologous to *A. thaliana* genes, were screened by searching against the BnIR database [31]. The ‘ZS11’ reference genome includes 150 *Bna.BGLU* genes, 219 *Bna.PRX* genes, and 59 *Bna.LAC* genes.

In our analysis, we identified a total of 4186 DEGs (1840 up-regulated and 2346 down-regulated) in the OrB8P vs. OrB5P comparison, 2667 DEGs (1515 up-regulated and 1152 down-regulated) in the OrS1P vs. OrB8P comparison, and 1418 DEGs (849 up-regulated and 569 down-regulated) in the OrS3P vs. OrS1P comparison. The DEGs were screened based on an adjusted *p*-value of <0.001 and an absolute FC > 4. Interestingly, 49 of these DEGs were common to all comparisons (Figure S5A). Additionally, we observed the differential expression of 22 *Bna.BGLU* genes, 16 *Bna.PRX* genes, and 0 *Bna.LAC* genes (Figure S5B–D). Furthermore, 18 *BGLU* genes and 11 *PRX* genes exhibited an FPKM value > 5. The significantly up-regulated genes *BnaA09.BGLU7* (*BnaA09G0566300ZS*), *BnaC04.BGLU10* (*BnaC04G0260400ZS*), *BnaA09.PRX31* (*BnaA09G0041100ZS*), *BnaA01.PRX47* (*BnaA01G0045400ZS)*, *BnaC01.PRX47* (*BnaC01G0051700ZS*), and *BnaA07.PRX64* (*BnaA07G0035700ZS*) may be responsible for decreased anthocyanin accumulation in ‘OrP’ petals.

Three main factors are likely responsible for the fading of ‘OrP’ petals (Figure 7). First, decreased anthocyanin biosynthesis may be linked to the down-regulated expression of the genes responsible for anthocyanin biosynthesis and the up-regulated expression of negative regulatory genes such as *BnaC06.MYBL2*. Second, the reduced relative anthocyanin content may be linked to the up-regulation of the genes involved in anthocyanin degradation. Third, as the petals develop, their surface area gradually increases, which dilutes the relative anthocyanin content.

The environmental factors such as strong light and high temperature may also contribute to the decomposition of anthocyanin [63]. The influence of light intensity on the petal cells may still be limited when the petals are wrapped by sepals before the buds open, and the degradation of anthocyanins after synthesis may be relatively small, resulting in a continuous increase in the relative concentration of anthocyanins and the Δa chromaticity value in the petals, showing a redder color. As the petals extend, when the petals are in the S1, S2, S3, and S4 stages, the effect of light intensity and duration on the petal cells on the upper surface gradually increases, resulting in an increase in the degradation of anthocyanins in the petals, making the petals appear to be a lighter red color. On the other hand, the increased temperature can also promote the degradation of anthocyanins and inhibit the expression of genes related to anthocyanin synthesis; indeed, these could also be the possible reasons to account for the following phenomena: at the end of February each year (about 10 °C), ‘OrP’ is in the early flowering period and the petals in S1 stage show a particularly bright red color, while the ‘OrP’ petals in S1 stage become noticeably lighter in mid-March (about 25 °C) during the exuberant flowering period. However, further experiments will be required to confirm these hypotheses.

In future research, breeders could create more brightly colored rapeseed flowers through genetic engineering technology according to the genes and methods identified in this study, such as, knocking out the *BnaMYBL2* gene to suppress the repressive effect on anthocyanin synthesis, and knocking out one or more ADE genes to suppress anthocyanin degradation by gene editing. Most importantly, it may be necessary to identify the key factors that inhibit the expression of *BnaA07.PAP2*, which is the key gene to ensure sustained anthocyanin synthesis and the relative concentration of anthocyanins in the petals, resulting in long lasting and brightly colored flower petals in *B. napus*.

## 5. Conclusions

In this work, metabolomic and transcriptomic analyses revealed the significant differences in the concentrations of anthocyanin glycoside derivatives and the expression of anthocyanin-related genes between ‘OrP’ and ‘ZS11’ petals. The orange-red coloration of ‘OrP’ petals resulted from a combination of cyanidin and paeoniflorin derivatives and carotenoids. We identified 49 differently expressed structural genes and 39 TFs that may play roles in anthocyanin biosynthesis and regulation, which may collectively participate in regulating the relative content of anthocyanins to affect the color of ‘OrP’ petals. In summary, this study helps to clarify the transcriptional regulation of anthocyanin biosynthesis and degradation in *B. napus* petals and offers useful genetic resources for breeding ornamental rapeseed cultivars.

## Data Availability

The transcriptomic data used in this study have been deposited in the GSA database under BioProject Accession Number PRJCA001841.

## Supplementary Materials

The following supporting information can be downloaded at: https://www.mdpi.com/article/10.3390/biology14060693/s1, Figure S1: The chromaticity values, chlorophyll contents of the ‘OrP’ and ‘ZS11’ petals, and surface area of the ‘OrP’ petals; Figure S2: Venn diagram of the differentially accumulated metabolites in different petal stages of ‘OrP’ and ‘ZS11’; Figure S3: Analysis of network topology for various soft thresholding (power value). Figure S4: Co-expression modules identified by WGCNA and the co-expression network of the anthocyanin biosynthetic process; Figure S5: The number of DEGs and anthocyanin degradation enzyme genes in ‘OrP’ petals at different stages. Table S1: List of primers for real-time PCR amplification; Table S2: Analysis of anthocyanin constituents in ‘OrP’ petals at stages S2 and S4 compared with ‘ZS11’; Table S3: Analysis of carotenoid constituents in ‘OrP’ petals at stages S2 and S4 compared with ‘ZS11’; Table S4: Differential metabolite analysis in ‘OrP’ and ‘ZS11’ petals at four different stages; Table S5: The main characteristics of cDNA libraries and RNASeq data of ‘OrP’ petals; Table S6: The DEGs in the comparison group of ‘OrP’ vs. ‘ZS11’; Table S7: Trails of ‘OrP’ and ‘ZS11’ petals for the gene co-expression network analysis; Table S8: Biological process in Gene Ontology analysis with all genes of each module from the gene co-expression network analysis; Table S9: KEGG analysis with all genes of each module from the gene co-expression network analysis.

## Abbreviations

The following abbreviations are used in this manuscript:

DEG	Differentially expressed gene
WGCNA	Weighted Gene Co-expression Network Analysis
TF	Transcription factor
TE	Transposable element
CCD	Carotenoid cleavage dioxygenase
ZEP	Zeaxanthin epoxidase
PAP	Production of anthocyanin pigment
RNA-Seq	Transcriptome sequencing
FPKM	Fragments Per Kilobase Million
FC	Fold change
GO	Gene Ontology
KEGG	Kyoto Encyclopedia of Genes and Genomes
EBGs	Early biosynthetic genes
LBGs	Late biosynthetic genes
MBW	MYB-bHLH-WD40
ADEs	Anthocyanin degradation enzymes
BGLU	β-glucosidase
PRX, POD, or PER	Peroxidase
PPO	Polyphenol oxidase
LAC	Laccase
