# Supplementary material for: Combined Multi-Omics and Co-Expression Network Analyses Uncover the Pigment Accumulation Mechanism of Orange-Red Petals in Brassica napus L
Source: Biology (Basel). 2025 Jun 13;14(6):693. doi: 10.3390/biology14060693 (PMC12190104; doi:10.3390/biology14060693)
Supplement: Supplementary file 1 [file biology-14-00693-s001.zip › Supplementary Figure S1-S5.V7-revised.pdf]

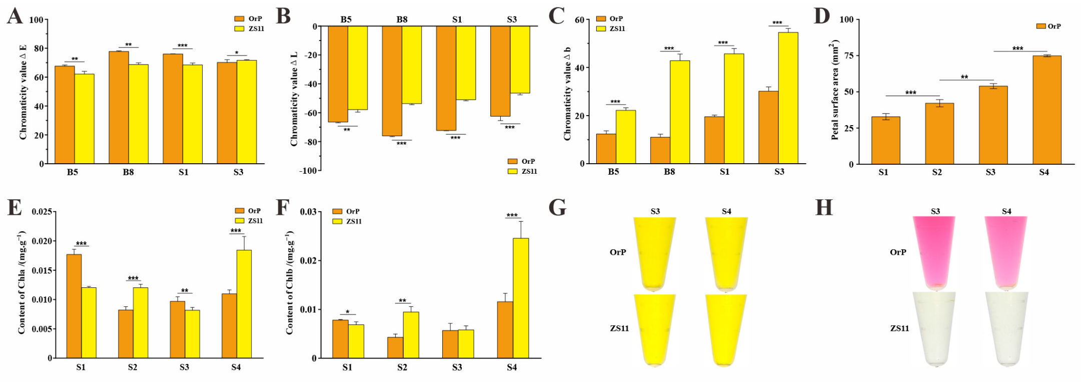

Supplementary Figure S1. The chromaticity values, chlorophyll contents of the 'OrP' and 'ZS11' petals, and surface area of the 'OrP' petals. (A-C) The chromaticity values of the petals in different stages for 'OrP' and 'ZS11'. The colorimetric values ΔE, ΔL, and Δb represent the total color, luminous-dark color, and yellow-blue color difference, respectively. (D) The surface area of 'OrP' petals from S1 to S4 stages. (E-F) The chlorophyll a and b contents of the petals in different stages for 'OrP' and 'ZS11'. (G-H) The extraction solution color difference of the petals in different stages for 'OrP' and 'ZS11'. Error bars indicated the standard deviation of three independent replicates. Asterisks (*, ** or ***) denoted significant differences at $P < 0.05$, $P < 0.01$, and $P < 0.001$, respectively.

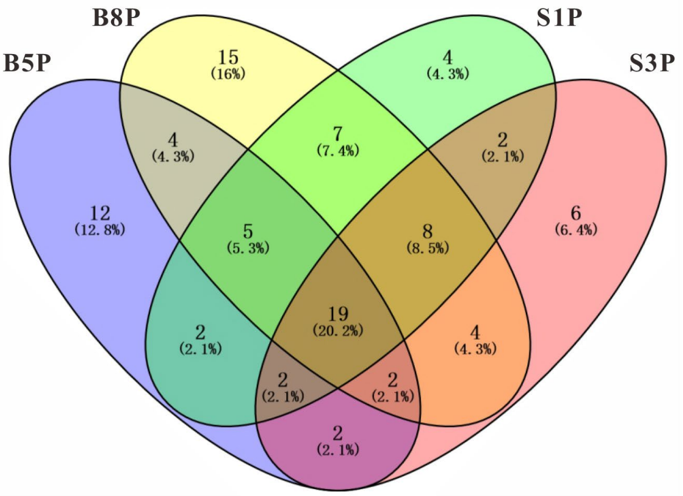

Supplementary Figure S2. Venn diagram of the differentially accumulated metabolites in different petal stages of 'OrP' and 'ZS11'.

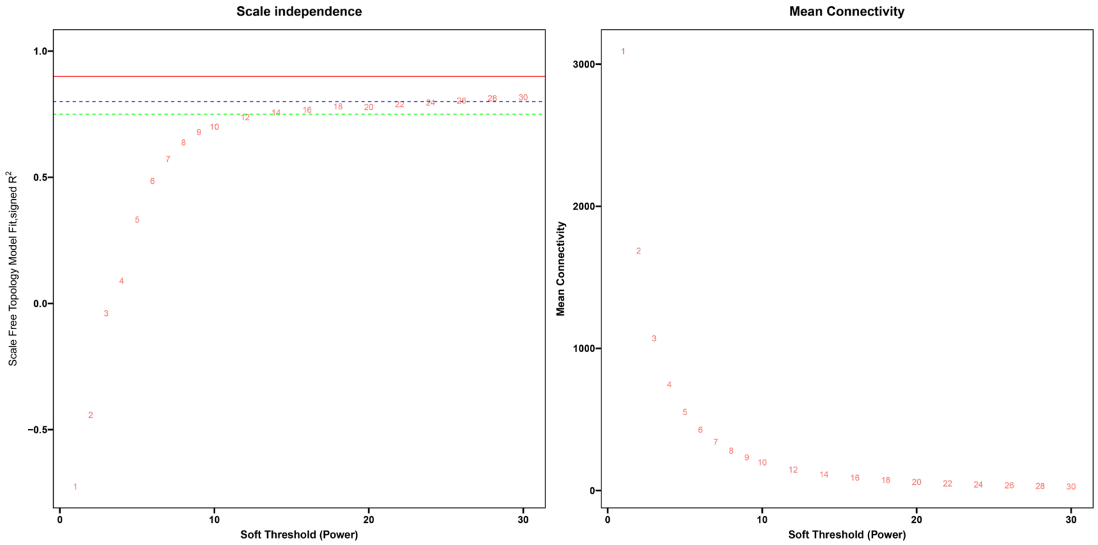

Supplementary Figure S3. Analysis of network topology for various soft thresholding (power value). The red solid line indicates a value of 0.9, blue dashed line indicates 0.8, and green dashed line indicates 0.75.

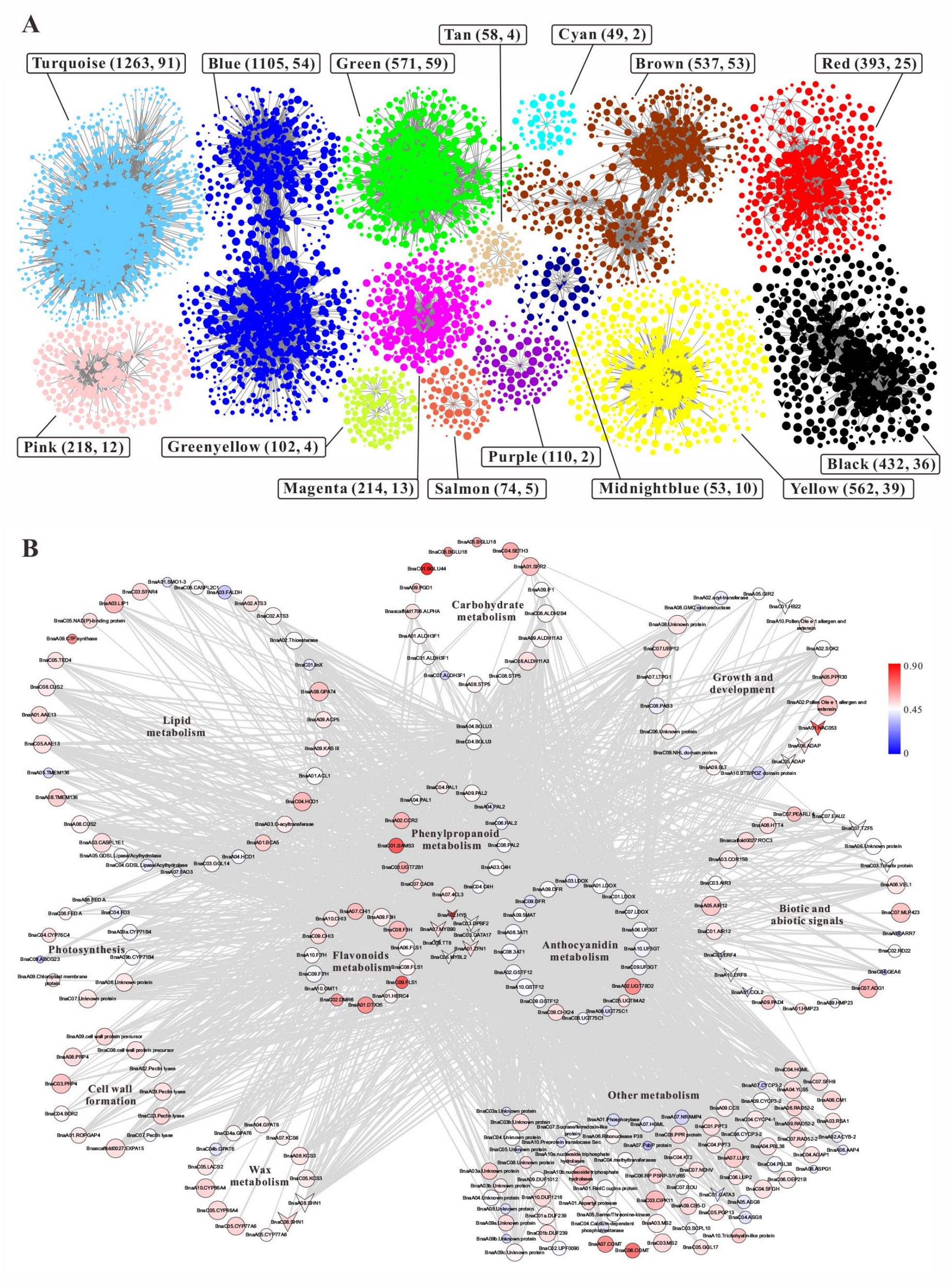

Supplementary Figure S4. Co-expression modules identified by WGCNA and the co-expression network of the anthocyanin biosynthetic process. (A) Visualization of the co-expression networks of the genes in each WGCNA module is done using Cytoscape. Total gene numbers and the number of transcription factor genes in each module are written in parentheses. (B) The Co-expression network of genes among the anthocyanin biosynthesis and other corresponding metabolism based on the RNA-seq DEGs in the KEGG pathway. Color of gene node indicates the gene trait significance, redder colors indicate greater significance. The size of the gene node represents the connectivity of a gene in the co-expression network. The edge thickness with gray line represents the weight value between nodes. The structural genes are drawn as circles, and the transcription

factor genes are drawn as chevrons.

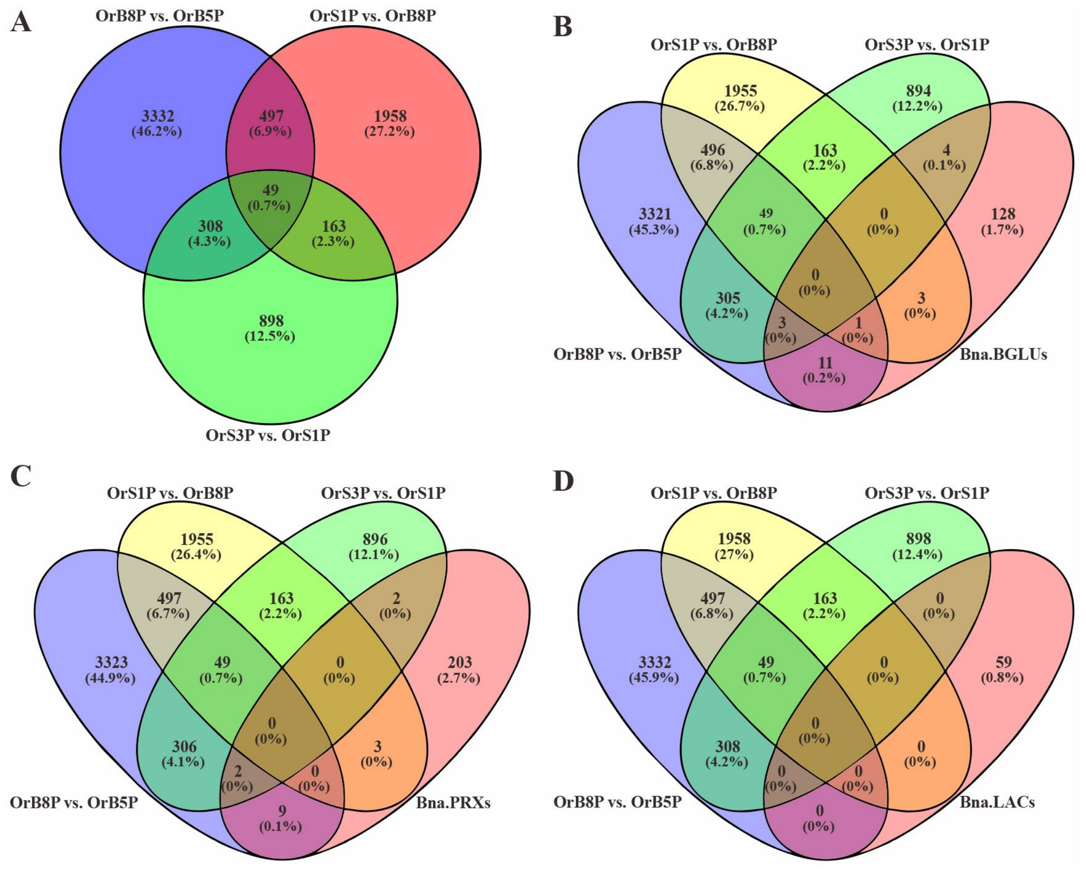

Supplementary Figure S5. The number of DEGs and anthocyanin degradation enzyme genes in 'OrP' petals at different stages. (A) Venn diagram showing overlapping DEGs between different comparison groups of OrP petals at different stages. (B-D) Venn diagram showing overlapping genes of the 'OrP' DEGs and the anthocyanin degradation enzyme genes, *Bna.BGLUs*, *Bna.PRXs*, *Bna.LACs*, respectively.
